# Variations in Cardiovascular Structure, Function, and Geometry in Midlife Associated With a History of Hypertensive Pregnancy

**DOI:** 10.1161/HYPERTENSIONAHA.119.14530

**Published:** 2020-04-20

**Authors:** Henry Boardman, Pablo Lamata, Merzaka Lazdam, Ashley Verburg, Timo Siepmann, Ross Upton, Amy Bilderbeck, Rhys Dore, Clare Smedley, Yvonne Kenworthy, Yrsa Sverrisdottir, Christina Y.L. Aye, Wilby Williamson, Odaro Huckstep, Jane M. Francis, Stefan Neubauer, Adam J. Lewandowski, Paul Leeson

**Affiliations:** 1 From the Oxford Cardiovascular Clinical Research Facility, Division of Cardiovascular Medicine, Radcliffe Department of Medicine (H.B., M.L., A.V., R.U., A.B., R.D., C.S., Y.K., C.Y.L.A., W.W., O.H., A.J.L., P. Leeson), University of Oxford, United Kingdom; 2 Nuffield Department of Surgical Sciences (Y.S.), University of Oxford, United Kingdom; 3 Nuffield Department of Women’s and Reproductive Health (C.Y.L.A.), University of Oxford, United Kingdom; 4 Oxford Centre for Clinical Magnetic Resonance Research (J.M.F., S.N.), University of Oxford, United Kingdom; 5 Department of Biomedical Engineering, King’s College London, United Kingdom (P. Lamata); 6 Department of Neurology, University Hospital Carl Gustav Carus, Technische Universität Dresden, Germany (T.S.); 7 Mohammed Bin Rashid University of Medicine, Dubai, UAE (Y.S.).

**Keywords:** blood pressure, echocardiography, hypertension, magnetic resonance imaging, pre-eclampsia, pregnancy, women

## Abstract

Hypertensive pregnancy is associated with increased maternal cardiovascular risk in later life. A range of cardiovascular adaptations after pregnancy have been reported to partly explain this risk. We used multimodality imaging to identify whether, by midlife, any pregnancy-associated phenotypes were still identifiable and to what extent they could be explained by blood pressure. Participants were identified by review of hospital maternity records 5 to 10 years after pregnancy and invited to a single visit for detailed cardiovascular imaging phenotyping. One hundred seventy-three women (age, 42±5 years, 70 after normotensive and 103 after hypertensive pregnancy) underwent magnetic resonance imaging of the heart and aorta, echocardiography, and vascular assessment, including capillaroscopy. Women with a history of hypertensive pregnancy had a distinct cardiac geometry with higher left ventricular mass index (49.9±7.1 versus 46.0±6.5 g/m^2^; *P*=0.001) and ejection fraction (65.6±5.4% versus 63.7±4.3%; *P*=0.03) but lower global longitudinal strain (−18.31±4.46% versus −19.94±3.59%; *P*=0.02). Left atrial volume index was also increased (40.4±9.2 versus 37.3±7.3 mL/m^2^; *P*=0.03) and E:A reduced (1.34±0.35 versus 1.52±0.45; *P*=0.003). Aortic compliance (0.240±0.053 versus 0.258±0.063; *P*=0.046) and functional capillary density (105.4±23.0 versus 115.2±20.9 capillaries/mm^2^; *P*=0.01) were reduced. Only differences in functional capillary density, left ventricular mass, and atrial volume indices remained after adjustment for blood pressure (*P*<0.01, *P*=0.01, and *P*=0.04, respectively). Differences in cardiac structure and geometry, as well as microvascular rarefaction, are evident in midlife after a hypertensive pregnancy, independent of blood pressure. To what extent these phenotypic patterns contribute to cardiovascular disease progression or provide additional measures to improve risk stratification requires further study.

## Abstract 2

Supplemental Digital Content is available in the text.

Vascular disorders, including cardiovascular disease and stroke, have become the largest cause of mortality and morbidity in women^1^ despite there being a unique sex-specific opportunity to identify those at high risk based on their pregnancy history. New onset hypertension during pregnancy,^2^ which occurs in up to 10% of women,^3^ is associated with a 4-fold increased risk of developing a range of cardiovascular disorders by midlife.^4^ However, identifying those who may gain the most from prevention advice after pregnancy is difficult as, for the majority, blood pressure falls^5^ and traditional risk assessment tools then underestimate risk.^6,7^

Hypertensive pregnancy is also associated with acute cardiovascular changes including left ventricular (LV) hypertrophy,^8,9^ LV systolic and diastolic impairment,^10^ arterial stiffening,^11^ and capillary rarefaction.^12^ Differences in these measures have been reported to still be identifiable for several years after a hypertensive pregnancy.^13,14^ If present in midlife, before the development of hypertension, these features could be useful risk markers,^15^ as they are independently associated with morbidity and mortality.^16–20^ However, the described phenotypes are also associated with changes in blood pressure, and any differences may reflect underlying development of hypertension rather than providing additional prognostic information.

Therefore, we recruited women 5 to 10 years after either a pregnancy complicated by hypertension or a normotensive pregnancy to undergo a comprehensive assessment of both cardiac and vascular structure, including personalized computational modeling of cardiac geometry. We used this multimodality data to identify whether, by midlife, any pregnancy-associated phenotypes were still identifiable and to what extent they could be explained by blood pressure.

## Methods

The data that support the findings of this study are available from the corresponding author upon reasonable request.

### Study Population and Pregnancy Data Collection

We studied women who had delivered a baby within the Oxford University Hospitals NHS Trust Maternity Unit between 5 and 10 years before the planned study visit (pregnancy between 1998 and 2010). Both women with a history of hypertensive pregnancy and women of equivalent age who gave birth during the same period after a normotensive pregnancy were recruited. Women with a history of hypertensive pregnancy included those with early onset preeclampsia (diagnosed before 34 weeks gestation), those with late onset preeclampsia (diagnosed at or after 34 weeks gestation), and those with gestational hypertension but not fulfilling the criteria for preeclampsia.^21^ Women who had delivered preterm but without a history of hypertension during pregnancy were identified and recruited to ensure comparable offspring birth gestations between those with a history of hypertension during pregnancy and the control group.

Maternity records for all participants were reviewed for every pregnancy registered at Oxford University Hospitals NHS trust. To ensure there were no additional pregnancies, which were registered and delivered at other hospitals, all participants completed a comprehensive questionnaire including details of all their pregnancies and whether they were complicated by hypertension or preterm delivery, data were also collected on current medications and smoking history. The study was approved by a local independent ethics committee (Oxfordshire Research Ethics Committee, reference 08/H0604/127), and participants provided signed informed consent.

Potential study recruits were identified after review of maternity medical records and study invitation letters sent. Those who responded were invited for a single study visit at the Cardiovascular Clinical Research Facility, University of Oxford, John Radcliffe Hospital, Oxford, United Kingdom.

### Cardiovascular Risk Assessment

Women attended a research clinic in the morning after a 12-hour fast and fasting blood samples were drawn. Blood pressure was recorded supine after a 10 minute rest. Central aortic blood pressure was estimated by applanation tonometry (SphygmoCor, AtCor Medical, West Ryde, Australia). At the end of the study, the participant was fitted with an ambulatory blood pressure device for 24 hours. Further details are available in the Data Supplement.

### Cardiac and Vascular Imaging and Analysis

#### Cardiovascular Magnetic Resonance

Cardiac structure and function were assessed using cardiovascular magnetic resonance (CMR; 1.5-T scanner and 12 channel body and spine array, Siemens, Munich, Germany) using previously described techniques.^22^ In brief, retrospectively ECG-gated steady state free precession images during breath hold were acquired of short-axis images of the left and right ventricles acquired at 1 cm intervals. Image analysis was performed using CVI42 version 5.1.1 (Circle Cardiovascular Imaging, Inc, Calgary, AB, Canada). Biventricular endocardial and epicardial borders were manually contoured at end-diastole and endocardial borders only in end-systole to measure ventricular volumes, ejection fraction, and myocardial mass.

#### Aortic Distensibility

Aortic structure and function were assessed at multiple levels using CMR (1.5-T scanner, Siemens, Munich, Germany) using previously described techniques.^23^ In brief, a retrospective ECG gated steady-state free precession sequence at end breath hold was acquired of the thoracic aorta at the level of the pulmonary artery bifurcation, both in the ascending and proximal descending aorta (AA and PDA, respectively); additional images were captured of the distal descending aorta 12 cm distal to the proximal descending aorta perpendicular to the lumen and the abdominal aorta at the level of the second lumbar vertebra (AbA). The maximal and minimum aortic area was measured using semiautomated edge detection algorithms (Matlab, Mathworks, Inc, MA). Compliance was calculated by dividing the change in aortic area by minimum aortic area. Distensibility was calculated by dividing compliance by central pulse pressure measured during the CMR scan using a brachial blood pressure cuff connected to an automated oscillometry device (Vicorder, Skidmore Medical, Taunton, United Kingdom). Global compliance and distensibility was calculated from the mean value of local measurements throughout the aorta.^24^

#### Pulse Wave Velocity

Pulse wave velocity was measured between carotid and femoral arteries.^25^ The distance was measured from sternal notch to left carotid pulse and sternal notch to left femoral pulse. Then, applanation tonometry (SphygmoCor; AtCor Medical, West Ryde, Australia) was used to obtain pressure waveforms of the carotid and femoral pulse, by holding the tonometer gently to partially compress the corresponding artery and record 10 consecutive pressure waveforms. A mean pressure waveform was calculated and the time difference from the R wave on the ECG recording to the pulse arrival time at each arterial measurement site used to derive the pulse wave velocity between the 2 sites.

#### Microvascular Assessment

Microvascular structure was assessed using intravital video capillaroscopy using methods previously described.^26^ In brief, a microscope and light emitting diode were used to image the dorsum of the middle phalanx of left hand and allow clear discrimination of capillaries filled with blood. Six distinct image fields were recorded for 1 minute each; then a small cuff was inflated at the base of the phalanx, to ensure capillary engorgement with blood and the same image fields recorded again. Image analysis was performed offline using Image ProPlus (MediaCybernetics, Rockville, MD). The average capillary density of each set of 6 fields was used to calculate functional and anatomic capillary density respectively.

#### Carotid Intima Media Measurement

Carotid intima media thickness was measured using previously described techniques.^27^ In brief, an ultrasound was performed of both left and right carotid arteries using a CX-50 ultrasound machine and L12-3 linear probe (Philips, Amsterdam, Netherlands). ECG gated cine loops were acquired of the common carotid artery 1 cm proximal to the bifurcation while the patient lay supine. Image analysis was performed offline using Brachial analyser version 5.6.15 (Medical Imaging Applications LLC, Coralville, IA) to measure the intima media thickness at end diastole.

### Cardiac Computational Models

A subgroup of 153 subjects were available for a detailed 3-dimensional computational analysis. The LV end diastolic anatomy was reconstructed from the contours using high order interpolation meshes as described in our previous work.^22,28^ The 153 meshes were then spatially aligned by their centre of mass, and by an orientation defined by their basal plane and the left to right direction set by the centre of mass of the LV and right ventricle. A statistical shape model was then built with a principal component analysis, finding the modes of anatomic variation of this cohort. The left ventricular meshes for the population have been made available to the scientific community (https://doi.org/10.6084/m9.figshare.11303306).

### Statistical Analysis

Statistical analysis was undertaken using Statistical Product and Service Solutions (SPSS) Version 20 (Armonk, NY). Normality was assessed using visual assessment of histograms and Shapiro-Wilk test. Comparison between 2 groups for continuous variables was performed using a 2-sided, independent-samples Student *T*-test (parametric data) and Mann-Whitney test (nonparametric data). Comparison between 2 groups for categorical variables was performed using a χ^2^ test. Multivariate linear regression models were performed using a forced entry method and unstandardized regression coefficients (β) reported. Multivariable models were also undertaken to compare if differences found between cohorts persisted after adjusting for other cardiovascular risk factors in addition to systolic blood pressure including age, body mass index, and total cholesterol:HDL. *P* values <0.05 were considered statistically significant. Modes of variation identified using principal component analysis for the 3-dimensional LV shape analysis were compared between groups using unpaired *T*-test. Previously reported differences in systolic blood pressure between women with a history of hypertensive compared with normotensive pregnancies were 10 mm Hg at a similar time after affected pregnancy [unpublished data]. The MESA (Multiethnic Study of Atherosclerosis),^29^ which reported associations between LV mass and blood pressure suggested that differences in LV mass of 4.8 gram would be expected with this difference in systolic blood pressure between groups. Seventy individuals in each group provided 80% power at *P*=0.01 to identify differences of this size between groups.

## Results

### Study Cohort Characteristics

One thousand five hundred seven women were identified who fulfilled International Society for the Study of Hypertension in Pregnancy criteria for preeclampsia or gestational hypertension or could be demonstrated to have had normotensive pregnancy, without preexisting cardiovascular or renal disease. Of these, 268 agreed to attend a study visit, of which 95 participated in a vascular-only imaging substudy, and 173 (73 with a history of normotensive pregnancy and 100 with a history of hypertensive pregnancy) underwent the full protocol and are reported in this paper. Table 1 describes the characteristics of women who had a history of hypertensive or normotensive pregnancy. Hypertensive pregnancy was associated with shorter gestation and lower birthweight, while overall these women had fewer but more complicated pregnancies. Smoking rates and body mass index were similar in both groups but hypertensive pregnancy was associated with lower HDL-cholesterol and raised HOMA-IR. Awake ambulatory blood pressures were not significantly different between groups. However, 9% of women with a history of hypertensive pregnancy were on antihypertensive medication. Women with a history of hypertensive pregnancy had higher nocturnal diastolic blood pressures, as well as higher office and central blood pressures.

**Table 1.**
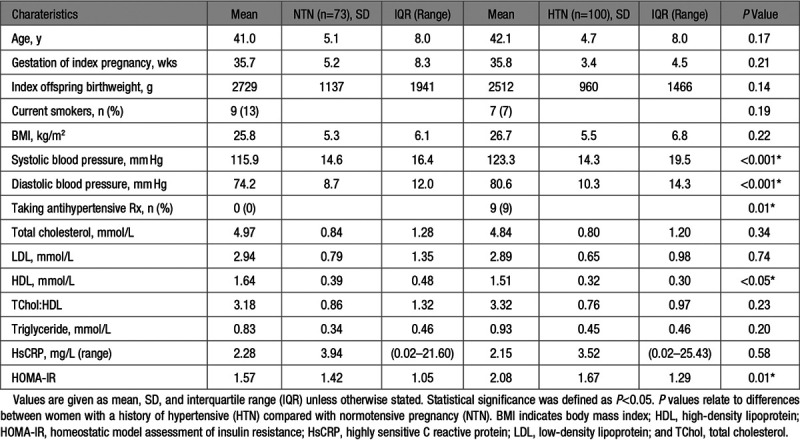
Characteristics of Cardiovascular and Pregnancy Factors in the Study Group

### Cardiac Structure

Women with a history of hypertensive pregnancy had relative LV hypertrophy (see Table 2) with higher LV mass (49.9±7.1 versus 46.0±6.5 g/m^2^, *P*=0.001) and mean wall thickness (5.5±0.9 versus 5.2±0.7 mm, *P*=0.02). RV mass was higher, though this did not reach statistical significance (15.6±3.2 versus 14.8±4.5 g/m^2^, *P*=0.06) and left atrial volume index was statistically higher (40.4±9.2 versus 37.3±7.3 mL/m^2^, *P*=0.03).

**Table 2.**
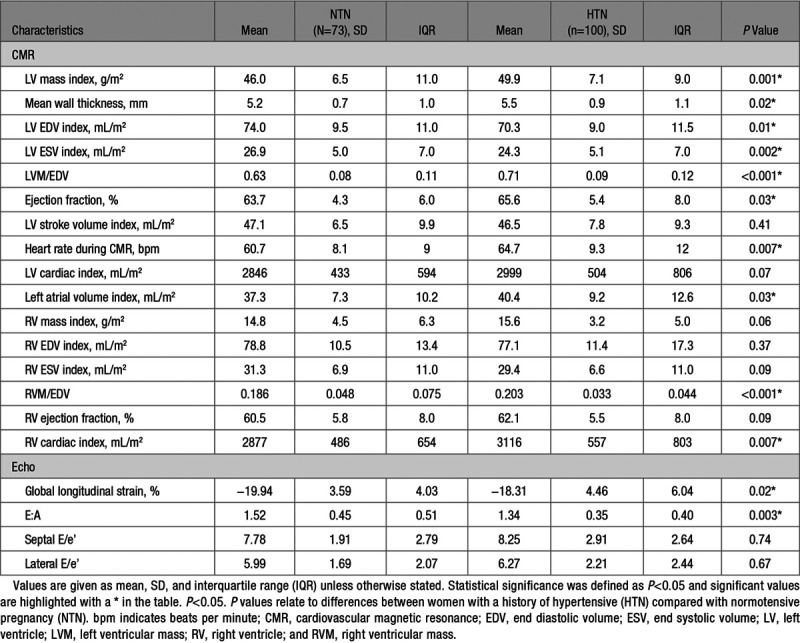
Cardiac Structure and Function

### Cardiac Function

Women with a history of hypertension during pregnancy had higher LV ejection fraction (65.6±5.4 versus 63.7±4.3 %, *P*=0.03) however there was no difference in stroke volume index (46.5±7.8 versus 47.1±6.5 mL/m^2^, *P*=0.41). LV global longitudinal strain was lower in those with a history of hypertensive pregnancy (-18.31±4.46 versus -19.94±3.59 %, *P*=0.02) as was E:A ratio (1.34±0.35 versus 1.52±0.45, *P*=0.003).

### Vascular Assessment

Aortic compliance and capillary density were lower in women with a history of hypertensive pregnancy (aortic compliance 0.240±0.053 versus 0.258±0.063, *P*=0.046; functional capillary density 105.4±23.0 versus 115.2±20.9 capillaries/mm^2^, *P*=0.01 respectively) (see Table 3).

**Table 3.**
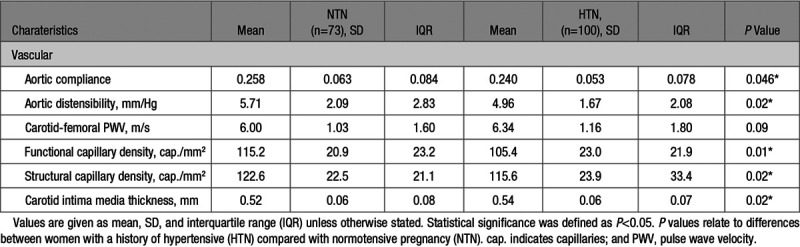
Vascular Structure and Function

### Adjustment for Blood Pressure

All cardiac and vascular differences which were statistically different between groups were adjusted to test whether differences remained after taking into account variation in blood pressure whether characterised as office or ambulatory blood pressure. Differences between groups in LV mass index, functional capillary density, LV end systolic volume index and left atrial volume index remained after adjustment for office blood pressure (using systolic or diastolic blood pressure - see supplementary material) and separately after adjustment for ambulatory blood pressure (using systolic or diastolic blood pressure - see supplementary material). No other cardiovascular differences present between groups were consistently present after adjustment for blood pressure.

### Additional Post Hoc Analysis

**Figure 1.**
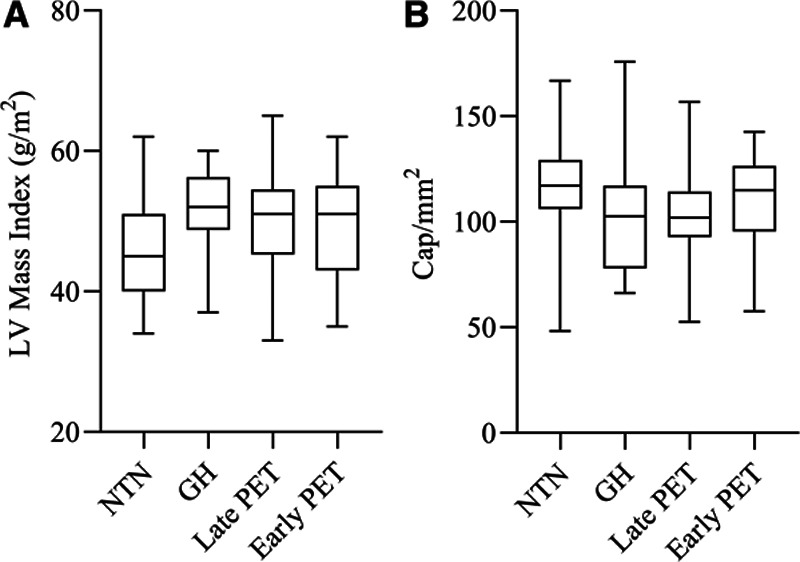
Variation in left ventricular mass and capillary density in midlife related to severity of hypertensive pregnancy. **A**, Represents left ventricular (LV) mass index in women with a history of normotensive pregnancy (NTN—45.9 g/m^2^) compared with women with a history of different hypertensive disorders during pregnancy: gestational hypertension (GH—51.4 g/m^2^; *P*<0.01), late onset preeclampsia (PET) (late PET—49.6 g/m^2^; *P*=0.01), and early onset preeclampsia (early PET—49.4 g/m^2^, *P*=0.02). **B**, Represents functional capillary density in women with a history of normotensive pregnancy (NTN—115 cap/mm^2^) compared with women with a history of different hypertensive disorders during pregnancy: (GH—103 cap/mm^2^; *P*<0.01), late onset preeclampsia (late PET—102 cap/mm^2^, *P*<0.01), and early onset preeclampsia (early PET—111 cap/mm^2^; *P*=0.40).

Additional adjustments, in multivariate models including age, BMI and total cholesterol:high density lipoprotein in addition to systolic blood pressure did not have a significant impact on the findings of cardiovascular phenotypic differences between groups. Posthoc analysis was undertaken to see if there was a “dose response” relationship between the severity of the hypertensive disorder of pregnancy and the cardiovascular phenotypic differences of LV mass index and functional capillary density. However, no consistent graded effect was observed across the different participant groups (Figure 1).

A sensitivity analysis was also undertaken to test whether differences in cardiac and vascular measures that persisted following adjustment by blood pressure were still present after exclusion of women who had a history of normotensive preterm delivery. Differences in LV mass index (45.8±6.0 versus 49.9±7.1 g/m^2^; *P*<0.01) and functional capillary density (119.8±17.2 versus 105.4±23.0 cap./mm^2^; *P*<0.01) remained, and were of similar magnitude, as were differences in LV end systolic volume index (27.7±4.7 versus 24.3±5.1 mL/m^2^; *P*=0.001); however, differences in left atrial volume index were no longer significant (37.6±7.5 versus 40.4±9.2 mL/m^2^; *P*=0.11).

A separate sensitivity analysis was undertaken to assess whether differences remained after exclusion of women taking antihypertensive medications at the time of the study visit. Differences in LV mass index, functional capillary density, LV end systolic volume index, and left atrial volume index all remained (*P*=0.001, *P*=0.01, *P*<0.01, and 0.03, respectively). A sensitivity analysis excluding participants who were current smokers at the time of the study demonstrated that the significant differences in functional capillary density remained.

### Shape Changes in the Left Ventricle

**Figure 2.**
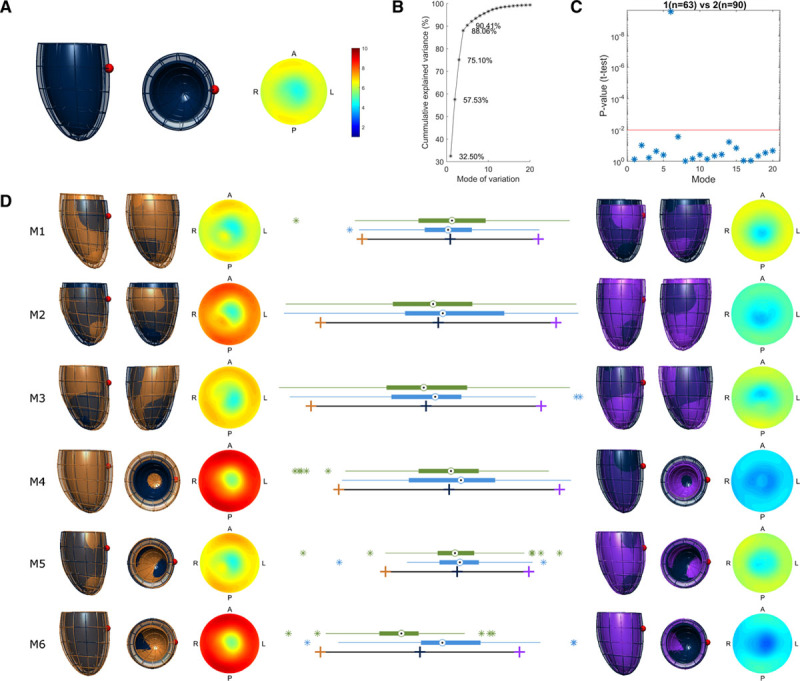
Major patterns of variation in left ventricular geometry in women in midlife and identification of those related to hypertensive pregnancy. **A**, Average anatomy (ie, atlas) of the 153 cases under study—2 views of the same 3-dimensional model in dark blue with a red sphere indicating the location of the right ventricle, and a thickness map (thickness colour map consistent throughout this figure). **B**, Cumulative variance explained by each mode of variation—mode 6 accounts only for the 1.7% of the variance in shape. **C**, Comparison of differences in shape between hypertensive and normotensive groups in the 20 first modes of variation (*P* value of unpaired *T*-test). **D**, The first 6 independent modes of variation. In all 6 cases, box-plots represent the hypertensive (green) and normotensive (blue) distributions in each model, with the average at coordinate 0 and is represented by a dark blue cross. In the 3-dimensional models, the average is again the dark blue left ventricular (LV) mesh overlaid on the orange and purple meshes. The orange cross and orange LV mesh represent −3 SD for each mode of variation; the purple cross and purple LV mesh, +3 SD for each mode of variation. As illustrated here, the modes of variation represent changes in the following: 1=ventricular length; 2=ventricular length linked to apex shift in the right to left direction and wall thickness; 3=apex shift in the right to left direction, linked to a milder wall thickness change; 4=wall thickness linked to diameter changes; 5=wall thickness with a proportion of apex position; and 6=wall thickness.

Statistical shape models were used to explore differences in shape with respect to an average anatomy (illustrated in Figure 2A). Within the whole group of women, 6 modes of variation accounted for 92% of the variance (Figure 2B), and these modes are shown in decreasing order of the amount of variance explained in Figure 2D. Visual assessment of the anatomy described by each mode identified that modes 1 and 2 describe LV length variation, modes 2, 3, and 5 apical orientation and modes 4 and 5 variation in wall thickness related to diameter. Only mode 6 varied significantly between women with a history of hypertensive pregnancy compared with normotensive pregnancy (Figure 2C and 2D). Mode 6 describes a specific change in LV wall thickness, without change in length, apical orientation, or basal diameter. Differences between groups in this mode remained after adjustment for blood pressure. Further subgroup analysis according to severity of hypertension during index pregnancy demonstrated this specific shape change in all pregnancy hypertension groups, without evidence of a dose response with severity of the pregnancy complication.

## Discussion

This study shows that, in midlife, women with a history of hypertensive pregnancy are more likely to have higher left ventricular mass index and left atrial volume index, as well as capillary rarefaction, independent of their individual blood pressure levels at that time. These differences remained statistically significant whether adjustment for blood pressure was performed with office or ambulatory measures. Furthermore, a distinct pattern of left ventricular mass distribution was identifiable using a 3-dimensional computational model, which was specifically related to a hypertensive pregnancy history.

Increased left ventricular mass is known to strongly associate with mortality of any cause, with women who have left ventricular hypertrophy being at greater risk than men.^16^ This risk extends into the normal range of left ventricular mass^30^ and persists in the absence of hypertension. In the MESA, a CMR-based study of men and women free of cardiovascular disease, a 10% increase in left ventricular mass correlated with a 40% increased risk of heart failure events.^31^ In our study, we found a 4 g/m^2^ difference in left ventricular mass index, comparable to an 8% difference, was evident between groups. This is consistent with previous reports that those who have had a hypertensive pregnancy have significantly greater left ventricular mass immediately postpartum.^10^ Scantlebury et al^32^ reported echo data in 2637 women showing that left ventricular mass was also significantly different >25 years after a hypertensive pregnancy. Despite the women, we studied with a history of hypertensive pregnancy had significantly higher LV mass (49.9±7.1 g/m^2^) compared with those with a history of normotensive pregnancy, the majority of values were within ranges reported for healthy populations in the United Kingdom (42±7 g/m^2^).^33^ However, our analysis suggests this increased left ventricular mass after hypertensive pregnancy is not explained by differences in blood pressure and can occur before development of hypertension or overt cardiovascular disease.

Women with a history of hypertensive pregnancy also exhibited reduced dermal capillary density. We have previously shown an increase in cerebrovascular white matter changes in woman after hypertensive pregnancies^34^ suggesting microvascular differences may be present across several vascular beds. Increased systemic vascular resistance, thought to be predominantly due to capillary rarefaction, is also reported during hypertensive pregnancy,^12^ when it is associated with pathophysiological myocardial hypertrophic adaptation^10^ and is thought to precede development of hypertension in those with a hypertensive family history.^35,36^ The presence of capillary rarefaction in individuals with increased left ventricular mass is consistent with reports of reduced capillary density in several phenotypes of heart disease including diastolic heart failure^37^ and microvascular coronary disease.^38^

We undertook unbiased geometric analysis to assess whether subtle shape differences, not identified by 2-dimemsional analysis or global parameters, could further describe cardiovascular phenotypic differences associated with a history of hypertensive pregnancy. We previously developed this methodology of cardiac CMR atlas formation to identify unique cardiac shape differences in young adult life associated with preterm birth.^22^ Since then, the techniques have been tried in multiple different settings including to demonstrate an association between LV hypertrophy and fat mass^39^ while Gilbert et al^40^ using a large cohort from the UK Biobank has demonstrated a stronger association between cardiovascular risk factors and cardiac atlas shapes than simple measures of mass and volume. In the current study, we identified that a global increase in LV wall thickness, independent of differences in blood pressure, is the main cardiac morphological difference between women with normotensive and hypertensive pregnancies, consistent with our findings of increased LV mass index and increased average wall thickness on mid ventricular 2-dimensional measurement. Whether the characteristic 3-dimensional variation is identifiable in other hypertensive disease cohorts, its use as a biomarker provides additional prognostic information to simple mass measures or whether the anatomic variation has specific functional impacts requires further study and longitudinal follow-up.

Interestingly, LV ejection fraction was slightly higher in those with a history of hypertensive pregnancy; however, global longitudinal strain was lower. This difference in ejection fraction appears to relate to the fact that stroke volume was similar between groups, but LV end diastolic volume was smaller in those with a history of hypertensive pregnancy.

Differences seem to be present throughout the spectrum of hypertensive disorders of pregnancy, as currently defined by guidelines, and our data does not support a dose response relationship between the timing of hypertension diagnosis in pregnancy with cardiovascular differences 9 years later. Although previous data^41^ has suggested a correlation between severity of hypertensive disorder at the time of pregnancy and later cardiovascular disease, it maybe that there are other, as yet unidentified, pregnancy or nonpregnancy related factors, which are important in identifying those most likely to exhibit phenotypic differences in later in life.

It is recognized that women with a history of spontaneous preterm delivery uncomplicated by hypertension have an elevated cardiovascular risk in later life.^42^ Hence, we purposefully recruited individuals with preterm delivery both with and without hypertension so that we could identify changes in cardiovascular phenotype specific to pregnancy hypertension rather than other pregnancy complications. It maybe that due to this aspect of study design, our results underestimate differences between those with a history of hypertensive pregnancy and a population with solely uncomplicated pregnancies. However, a sensitivity analysis was performed excluding those with a normotensive preterm delivery and the main findings of differences in LV mass, capillary rarefaction, and LV size remained.

Our study has some important limitations. First, the depth of multimodality imaging across multiple vascular and cardiac phenotypes combined with the detailed extraction and adjudication of pregnancy history from medical records meant the group sizes are relatively small. Therefore, we may have been underpowered to identify subtle differences between certain imaging phenotypes, subgroups, or severities of hypertensive pregnancy. Second, the study was performed at a single time point several years after delivery and causality cannot be inferred. The particular time point chosen provides confidence that any differences detected are independent of transient physiological changes of pregnancy and precede development of established hypertension but longitudinal studies, ideally extending from prepregnancy to later life will be of value to establish the time course of changes. Furthermore, understanding links between these phenotypes and disease in later life will be of value to determine whether it is possible to identify high-risk individuals using imaging earlier in life.

## Perspectives

Our findings demonstrate that hypertensive disorders of pregnancy are associated with structural differences in the heart and microvasculature in midlife, independent of blood pressure. Hypertension during pregnancy is now a well-recognized risk factor for cardiovascular disease in later life.^43^ Left ventricular hypertrophy is a risk factor for heart failure^44^ and, independent of hypertension, is associated with mortality in women more than in men.^16^ Microvascular rarefaction is associated with heart failure^37^ and angina.^45^ Therefore, the differences identified in this study require further evaluation as possible biomarkers to identify women at high risk of later cardiovascular disease. If so, future interventions targeting women expressing these surrogate markers may be a valid prevention strategy.

## Sources of Funding

This work was supported by grants to Professor Leeson from the British Heart Foundation (FS/06/024 and FS/11/65/28865), National Institute for Health Research Oxford Biomedical Research Centre and Oxford British Heart Foundation Centre for Research Excellence. T. Siepmann was supported by a European Academy of Neurology Fellowship and received grants from the Michael J. Fox Foundation and Prothena Biosciences. P. Lamata holds a Wellcome Trust Senior Research Fellowship (209450/Z/17/Z). A. Bilderbeck received salaries from P1vital Ltd.

## Disclosures

None.

## Supplementary Material



## Novelty and Significance

### What Is New?

- Prior studies have shown that women have cardiovascular phenotypic changes peripartum and are at increased risk of cardiovascular disease in later life.
- However which phenotypic changes persist long term and whether these changes are explained by differences in blood pressure is unknown.

### What Is Relevant?

- Hypertensive disorders of pregnancy are common, occurring in up to 10% of women.
- Typically hypertension resolves postpartum; however, cardiovascular risk remains elevated and there may be disease specific mechanisms, which are distinct from essential hypertension.

### Summary

Our findings demonstrate relative LV hypertrophy and capillary rarefaction several years after hypertensive pregnancy. These differences are not explained by differences in blood pressure.

Traditional assessment tools relying on blood pressure may underestimate cardiovascular risk in women with a history of hypertensive pregnancy.
