# Supplementary material for: Variations in Cardiovascular Structure, Function, and Geometry in Midlife Associated With a History of Hypertensive Pregnancy
Source: Hypertension. 2020 Apr 20;75(6):1542–50. doi: 10.1161/HYPERTENSIONAHA.119.14530 (PMC7682801; doi:10.1161/HYPERTENSIONAHA.119.14530)
Supplement: Supplementary file 1 [file hyp-75-1542-s001.docx]

# SUPPLEMENTAL MATERIAL

**Variations in cardiovascular structure, function and geometry in mid-life associated with a history of hypertensive pregnancy**

**Boardman: Cardiovascular phenotype after hypertensive pregnancy**

Henry BOARDMAN^a^, DPhil, Pablo LAMATA^b^, PhD, Merzaka LAZDAM^a^, DPhil, Ashley VERBURG^a^, Timo SIEPMANN^c^, MD, Ross UPTON^a^, Amy BILDERBECK^a^, DPhil, Rhys DORE^a^, BA, Clare SMEDLEY^a^, BA, Yvonne KENWORTHY^a^, BSc, Yrsa SVERRISDOTTIR^d,e^, PhD, BSc, Christina Y. L. AYE^a,f^, DPhil, Wilby WILLIAMSON^a^, MSc, MRCP, Odaro HUCKSTEP^a^, DPhil, Jane M. FRANCIS^g^, DCR(R), Stefan NEUBAUER^g^, MD, FRCP, Adam J LEWANDOWSKI^a^, DPhil, Paul LEESON^a^ PhD, FRCP*

a. Oxford Cardiovascular Clinical Research Facility, Division of Cardiovascular Medicine, Radcliffe Department of Medicine, University of Oxford, Oxford, UK.

b. Department of Biomedical Engineering, King's College London, London, UK.

c. Department of Neurology, University Hospital Carl Gustav Carus, Technische Universität Dresden, Dresden, Germany.

d. Nuffield Department of Surgical Sciences, University of Oxford, Oxford, UK.

e. Mohammed Bin Rashid University of Medicine, Dubai, UAE

f. Nuffield Department of Women’s and Reproductive Health, University of Oxford, Oxford, UK.

g. Oxford Centre for Clinical Magnetic Resonance Research, University of Oxford, Oxford, UK

*Correspondence and requests for reprints: Prof. Paul Leeson, Cardiovascular Clinical Research Facility, Division of Cardiovascular Medicine, Radcliffe Department of Medicine, University of Oxford, John Radcliffe Hospital, Oxford. OX3 9DU, UK. Tel: +44 1865 572846. E-mail: paul.leeson@cardiov.ox.ac.uk

**SUPPLEMENTAL METHODS**

**Biomarker collection**

Participants had blood collected whilst fasted. Methods have been previously described ([1](#_ENREF_1)9). In brief, samples were centrifuged at 3000 x G for 15 minutes at 40C. Serum, plasma and red cells were extracted and stored separately at -800C.

Quantikine enzyme-linked immunosorbent assays (ELISA) were performed to measure vascular endothelial growth factor A (VEGF-A), soluble fms-like tyrosine kinase-1 (sFLT-1) and soluble endoglin (sENG) concentrations. All ELISAs used colorimetric sandwich Quantikine technique (Quantikine, R & D Systems Europe, Abingdon, UK) and all samples, including standards, were plated in duplicate.

Optical density was measured at 450nm using a FLUOstar Omega microplate reader (BMG Labtech, KBioScience, USA) and the results analysed using Omega Data Analysis software (BMG Labtech, KBioScience, USA). Duplicate readings for each sample and standard were compared and those with a coefficient of variation >15% were repeated. Standard curves were calculated using a four-parameter logistic curve-fit.

**Heart rate variability**

In a subgroup, at the end of the study visit, participants were fitted with a holter monitor (Lifecard CF, Spacelabs, Snoqualmie, Washington, USA). A high resolution ECG (1024 Hz) was recorded continuously for 24 hours. The ECG recordings were analysed using Pathfinder SL version 1.7.1.4557 (Spacelabs, Snoqualmie, Washington, USA). Recordings were reviewed both manually and using automated techniques to identify and then remove artefact, ectopy and arrhythmia. To further ensure analysis of only normal beats, beat to beat intervals >120% or <80% of the previous interval were excluded, as these were more likely to represent artefact, ectopy or arrhythmia. Heart rate variability was measured for the duration of edited recording. Time and frequency domain analyses were measured over 5 minute epochs and averaged over the recording period. Frequency domain analyses were further fast Fourier transformed for each epoch to assess differences in frequency power bands.

**Exploratory analyses - menstrual data**

Information on menstrual period was collected using questionnaires and two additional post-hoc analyses were undertaken to assess the effect of menstrual cycle on the main vascular findings. The first adjusted for premenopausal or peri/postmenopausal status based on classification of participants’ in two groups according to whether they had their last menstrual period within 30 days (premenopausal) or more than 30 days (peri or post-menopausal) prior to the study visit. A second analysis was performed for those participants who reported their last menstrual period within the last 30 days based on number of days since their last menstrual period as a continuous variable.

**Exploratory analyses - pregnancy characteristics**

Additional exploratory correlation analysis was undertaken to examine relationships between cardiovascular phenotypic differences found to be significantly different between cohorts and relevant pregnancy characteristics of offspring birthweight and delivery gestation. To assess if a dose response was present cardiovascular phenotypic differences were also compared between those who had a history of one hypertensive pregnancy and those with a history of more than one. Multivariate linear regression models were performed with adjustment for age. P-values were deemed significant if <0.05.

Further sensitivity analysis was undertaken to assess if cardiovascular phenotypic differences remained when excluding those with a history of normotensive preterm delivery and those taking hypertensive medication at the time of the study.

**SUPPLEMENTAL RESULTS**

**Biomarkers**

There were no significant differences in angiogenic markers between groups (see S1).

**Heart rate variability**

There were no significant differences in heart rate variability between groups (see S2).

**Exploratory analyses - menstrual data**

In the reduced study population (n=104) who reported information on menstrual period, further adjustment for menopausal status and number of days since last menstrual period did not alter the finding of differences in functional capillary density between hypertensive and normotensive pregnancy groups; however, differences in aortic compliance, distensibility and carotid intima media thickness no longer were statistically different (all p>0.05).

**Exploratory analyses - pregnancy characteristics**

There were no significant correlations, in the entire study population and separately within only those with a history of hypertensive pregnancy, between LV mass index or functional capillary density and offspring birthweight or delivery gestation. There was also no significant difference between LV mass index or functional capillary density between those with a history of one hypertensive pregnancy and those with more than one (p=0.18 and p=0.94 respectively).

Sensitivity analysis excluding those with a history of normotensive preterm delivery and those taking hypertensive medication at the time of the study demonstrated the majority of the main findings remained (LV mass index: 45.8±6.0 vs 49.6±7.1g/m^2^, p=0.006; functional capillary density: 119.8±17.2 vs 105.2±22.6cap./mm^2^, p=0.002; LV ESV index: 27.7±4.7 vs 24.6±5.1ml/m^2^, p=0.002) though differences in left atrial volume index were no longer significant (37.6±7.5 vs 40.5±9.1ml/m^2^, p=0.10).

**S1: Biomarker results**

| Charateristics | NTN | HTN | p-value |
| --- | --- | --- | --- |
| sEndoglin (ng/ml) | 4.13±1.02 | 3.94±0.93 | 0.18 |
| sFLT1 (pg/ml) | 74.99±19.98 | 77.21±21.77 | 0.67 |
| VEGF (pg/ml) | 54.74±25.44 | 65.59±34.92 | 0.08 |
| sFLT: VEGF | 1.75±1.19 | 1.48±0.85 | 0.12 |
| Values as mean ± standard deviation. Bold text indicates significant group differences at p<0.05. Abbreviations: sFLT-1, soluble fms-like tyrosine kinase-1; VEGF-A, vascular endothelial growth factor A; sENG, soluble endoglin. | | | |

**S2: Heart rate variability**

| Characteristics | NTN  (n=49) | HTN  (n=49) | p-value |
| --- | --- | --- | --- |
| Recording characteristics |  |  |  |
| Recording length (hours) | 22.3±6.4 | 21.0±7.0 | 0.99 |
| Mean heart rate (beats/minute) | 76.9±9.7 | 79.8±9.8 | 0.20 |
| Heart rate variability |  |  |  |
| SDNN Index (msec) | 62.4±17.9 | 61.2±15.5 | 0.86 |
| RMSSD (msec) | 41.1±14.9 | 35.0±13.4 | 0.06 |
| SDSD (msec) | 31.0±12.7 | 26.3±11.0 | 0.07 |
| pNN50 (%) | 13.6±8.8 | 10.5±7.5 | 0.10 |
| VLF (msec^2^) | 1209±1016 | 998±755 | 0.20 |
| LF (msec^2^) | 992±926 | 892±776 | 0.33 |
| HF (msec^2^) | 652±494 | 564±592 | 0.14 |
| VHF (msec^2^) | 121±286 | 74±60 | 0.94 |
| LF: HF | 1.97±1.35 | 2.24±1.53 | 0.44 |
| Mean ± standard deviation, bold text indicates significant group differences at p<0.05; SDNN Index: standard deviation of NN intervals index; RMSSD: square root of the mean of the sum of the squares of differences between adjacent NN intervals; SDSD: standard deviation of differences between adjacent NN intervals; pNN50: proportion of adjacent NN intervals varying by 50 msec or more; VLF: very low frequency; LF: low frequency; HF: high frequency; VHF: very high frequency.  **S3. Cardiovascular differences with adjustment for office blood pressure**   \| Charateristics \| Adjusted for SBP \| \| Adjusted for DBP \| \| \| --- \| --- \| --- \| --- \| --- \| \| β \| p - value \| β \| p - value \| \| LV mass index (g/m^2^) \| 2.99 \| <0.01 \| 3.44 \| <0.01 \| \| LV mean wall thickness (mm) \| 0.15 \| 0.24 \| 0.17 \| 0.21 \| \| LV EDV Index (ml/m^2^) \| -3.34 \| 0.03 \| -2.63 \| 0.09 \| \| LV ESV Index (ml/m^2^) \| -2.37 \| <0.01 \| -2.31 \| <0.01 \| \| Left atrial volume index (ml/m^2^) \| 3.07 \| 0.04 \| 3.55 \| 0.02 \| \| LV ejection fraction (%) \| 0.02 \| 0.06 \| 0.02 \| 0.03 \| \| Global longitudinal strain (%) \| 1.10 \| 0.13 \| 0.80 \| 0.28 \| \| E:A \| -0.13 \| 0.04 \| -0.11 \| 0.09 \| \| Aortic compliance \| -0.007 \| 0.41 \| -0.007 \| 0.49 \| \| functional capillary density (cap./mm^2^) \| -10.3 \| 0.01 \| -10.8 \| <0.01 \| \| structural capillary density (cap./mm^2^) \| -8.6 \| 0.05 \| -8.3 \| 0.06 \| \| Carotid intima media thickness (mm) \| 0.01 \| 0.16 \| 0.02 \| 0.05 \|   Statistical significance was defined as p<0.05. *P-*values relate to differences between women with a history of hypertensive (HTN) compared to normotensive pregnancy (NTN). Abbreviations: cap., capillaries; LV, left ventricle; EDV, end diastolic volume; ESV, end systolic volume. | | | |

**S4. Cardiovascular differences with adjustment for ambulatory blood pressure**

| Charateristics | Adjusted for SBP | | Adjusted for DBP | |
| --- | --- | --- | --- | --- |
|  | β | p - value | β | p - value |
| LV mass index (g/m^2^) | 3.43 | **0.003** | 3.58 | **0.003** |
| LV mean wall thickness (mm) | 0.23 | 0.08 | 0.25 | 0.07 |
| LV EDVindex (ml/m^2^) | -3.74 | **0.02** | -3.43 | **0.03** |
| LV ESVindex (ml/m^2^) | -2.72 | **<0.01** | -2.63 | **<0.01** |
| Left atrial volume index (ml/m^2^) | 3.09 | **0.04** | 3.10 | **0.04** |
| LV ejection fraction (%) | 0.02 | **0.03** | 0.02 | **0.03** |
| Global longitudinal strain (%) | 1.29 | 0.08 | 1.37 | 0.07 |
| E:A | -0.17 | **0.01** | -0.16 | **0.01** |
| Aortic compliance | -0.014 | 0.14 | -0.013 | 0.16 |
| functional capillary density (cap./mm^2^) | -10.1 | **0.02** | -10.5 | **0.01** |
| structural capillary density (cap./mm^2^) | -7.3 | 0.09 | -7.9 | 0.07 |
| Carotid intima media thickness (mm) | 0.02 | 0.10 | 0.02 | 0.08 |

Statistical significance was defined as p<0.05. P-values relate to differences between women with a history of hypertensive (HTN) compared to normotensive pregnancy (NTN). Abbreviations: cap., capillaries; LV, left ventricle; EDV, end diastolic volume; ESV, end systolic volume.
