# Supplementary figures and images for: Variations in Cardiovascular Structure, Function, and Geometry in Midlife Associated With a History of Hypertensive Pregnancy
Source: Hypertension. 2020 Apr 20;75(6):1542–50. doi: 10.1161/HYPERTENSIONAHA.119.14530 (PMC7682801; doi:10.1161/HYPERTENSIONAHA.119.14530)

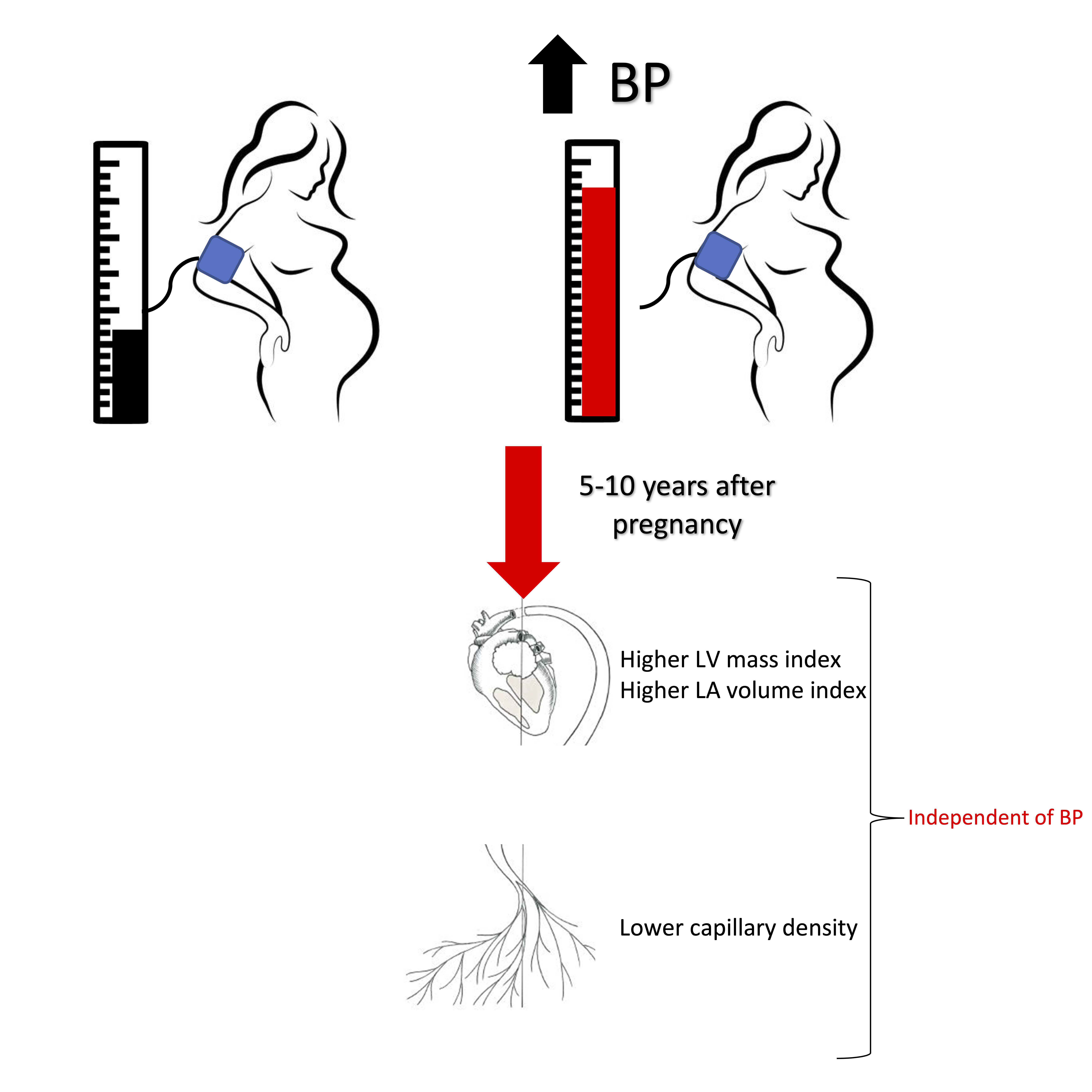

Supplement: Supplementary file 2 [file hyp-75-1542-s002.jpg]
